# Full-field, conformal epiretinal electrode array using hydrogel and polymer hybrid technology

**DOI:** 10.1038/s41598-023-32976-9

**Published:** 2023-04-28

**Authors:** Muru Zhou, Benjamin K. Young, Elena della Valle, Beomseo Koo, Jinsang Kim, James D. Weiland

**Affiliations:** 1 Macromolecular Science and Engineering Program, University of Michigan, Ann Arbor, 48105 USA; 2 Department of Ophthalmology, Oregon Health and Sciences University, Portland, OR 97239 USA; 3 Biomedical Engineering, University of Michigan, Ann Arbor, 48105 USA; 4 Chemical Engineering, University of Michigan, Ann Arbor, 48105 USA; 5 Materials Science and Engineering, University of Michigan, Ann Arbor, 48105 USA; 6 Chemistry, University of Michigan, Ann Arbor, 48105 USA; 7 Biointerfaces Institute, University of Michigan, Ann Arbor, 48105 USA; 8 Ophthalmology and Visual Sciences, University of Michigan, Ann Arbor, 48105 USA

**Keywords:** Biomaterials, Biomedical materials, Implants, Retinal diseases

## Abstract

Shape-morphable electrode arrays can form 3D surfaces to conform to complex neural anatomy and provide consistent positioning needed for next-generation neural interfaces. Retinal prostheses need a curved interface to match the spherical eye and a coverage of several cm to restore peripheral vision. We fabricated a full-field array that can (1) cover a visual field of 57° based on electrode position and of 113° based on the substrate size; (2) fold to form a compact shape for implantation; (3) self-deploy into a curvature fitting the eye after implantation. The full-field array consists of multiple polymer layers, specifically, a sandwich structure of elastomer/polyimide-based-electrode/elastomer, coated on one side with hydrogel. Electrodeposition of high-surface-area platinum/iridium alloy significantly improved the electrical properties of the electrodes. Hydrogel over-coating reduced electrode performance, but the electrodes retained better properties than those without platinum/iridium. The full-field array was rolled into a compact shape and, once implanted into ex vivo pig eyes, restored to a 3D curved surface. The full-field retinal array provides significant coverage of the retina while allowing surgical implantation through an incision 33% of the final device diameter. The shape-changing material platform can be used with other neural interfaces that require conformability to complex neuroanatomy.

## Introduction

Implantable neural stimulators prostheses are widely used to treat various neural disorders^1^. However, traditional electrode arrays use materials that are relatively rigid and may not conform well to soft neural tissues, which have anatomical curvatures. Thus, compression and shear forces created by the neural interface can damage the nerves and induce severe injury responses and the formation of fibrosis^2^. In addition to mechanical damage, increased distance from electrodes to the target tissue due to non-conformity can reduce stimulation efficiency and safety^3^. As a result, the need for a curved electrode array is essential for the retinal prosthesis to adapt to the spherical structure of the eye and provides a wide-field coverage to provide peripheral vision.

Visual prosthesis, one category of neural interfaces, can improve the quality of life of people with retinitis pigmentosa, a progressive disease resulting in mid-stage peripheral blindness and late-stage central blindness^4–6^. While central vision is essential for activities such as reading and recognizing objects and faces, the loss of peripheral vision can also greatly impair the mobility of patients^7^. However, commercial retinal prostheses have retinal arrays limited to 20 degrees of visual field or less. For example, the Argus II retinal array covers a 5x3 mm^2^ area of the retina which diagonally is approximately 20 degrees^8^. For safety reasons, the eye wall incision that enable implantation must be limited to roughly 5 mm. Increasing this incision length adds risk. This guideline limits the size of a retinal array. Experimental arrays of larger size (up to 14 mm diameter) can be folded for insertion, then unfolded to cover greater peripheral areas^9–13^ but require additional development before use in humans. Thus, there is a need to continue development of novel retinal arrays that incorporate new materials to meet the demands imposed by implantation (folded array for insertion through an eyewall incision) and function (large array to stimulate both central and peripheral retina).

Shape morphable neural interfaces are an increasingly attractive solution because of its ability to reconfigure into 3D shapes to conform to its target tissues^14–17^. Shape morphable materials, including shape memory polymers^18^ and hydrogels^19^, can not only provide shape reconfiguration responding to various stimuli but also may be packaged into a compact, folded state for implantation. Among those materials, hydrogels can be designed to respond to different stimuli, including temperature^20^ and moisture^21^, which are two stimuli that can indicate implantation within the body. Hydrogels can be fixed at an arbitrary shape by dehydration and recover to their hydrated shape after swelling. In addition, hydrogels can be very soft with an elastic modulus ranging from 1 to 100 kPa, and can be biocompatible depending on the compositions^22,23^, which make them a promising material for implantable electronics.

Hydrogels may lack the mechanical strength to support micro-patterned metal electrodes. A complete technical approach for a shape morphable retinal array can utilize polymers proven to provide mechanical strength and electrical insulation with a hydrogel layer to impose the desired shape. PDMS has good biocompatibility^24^ and has a low Young’s modulus compared with other materials used in neural interface fabrication^25^. There are multiple examples of using PDMS in flexible neural interfaces^26,27^. PDMS in combinations with other materials such as parylene C and polyimide have been explored as well^28–30^. Different research groups demonstrated the combination of hydrogel and PDMS to improve conformability^16,31^. However, use of hydrogel as the actuator to reconfigure electrode array into 3D shape has not been widely reported. In addition, different surface treatments have been explored to promote adhesion between the elastomer and hydrogel^32,33^. The longevity of such hybrid system in vivo demonstrated robustness up to 6 weeks^33^.

Here, we describe fabrication and testing of a full-field array (FFA), which can be incorporated into an epiretinal prosthesis. The FFA consists of a polyimide/PDMS polymer hybrid with gold circular microelectrodes, coated on one side with a polyacrylamide (PAAm) hydrogel. Polyimide supports conducting lines and electrode sites , while PDMS is the mechanical substrate and hydrogel interface. Platinum-iridium alloy (PtIr) was deposited on the electrode sites prior adding the hydrogel layer. We found that hydrogel deposition over the PtIr electrode sites increased impedance, but that the electrical properties remained sufficient to support electrical stimulation. Two prototype arrays were implanted ex vivo pig eyes. The final array spans 34 mm of retina, making FFA the largest epiretinal array demonstrated to date. Our findings support demonstrate important aspects of the technical approach, which if incorporated into a retinal prosthesis would provide substantially increased visual field compared to current clinical devices.

## Results

### Design and fabrication of full-field array (FFA)

**Figure 1:**
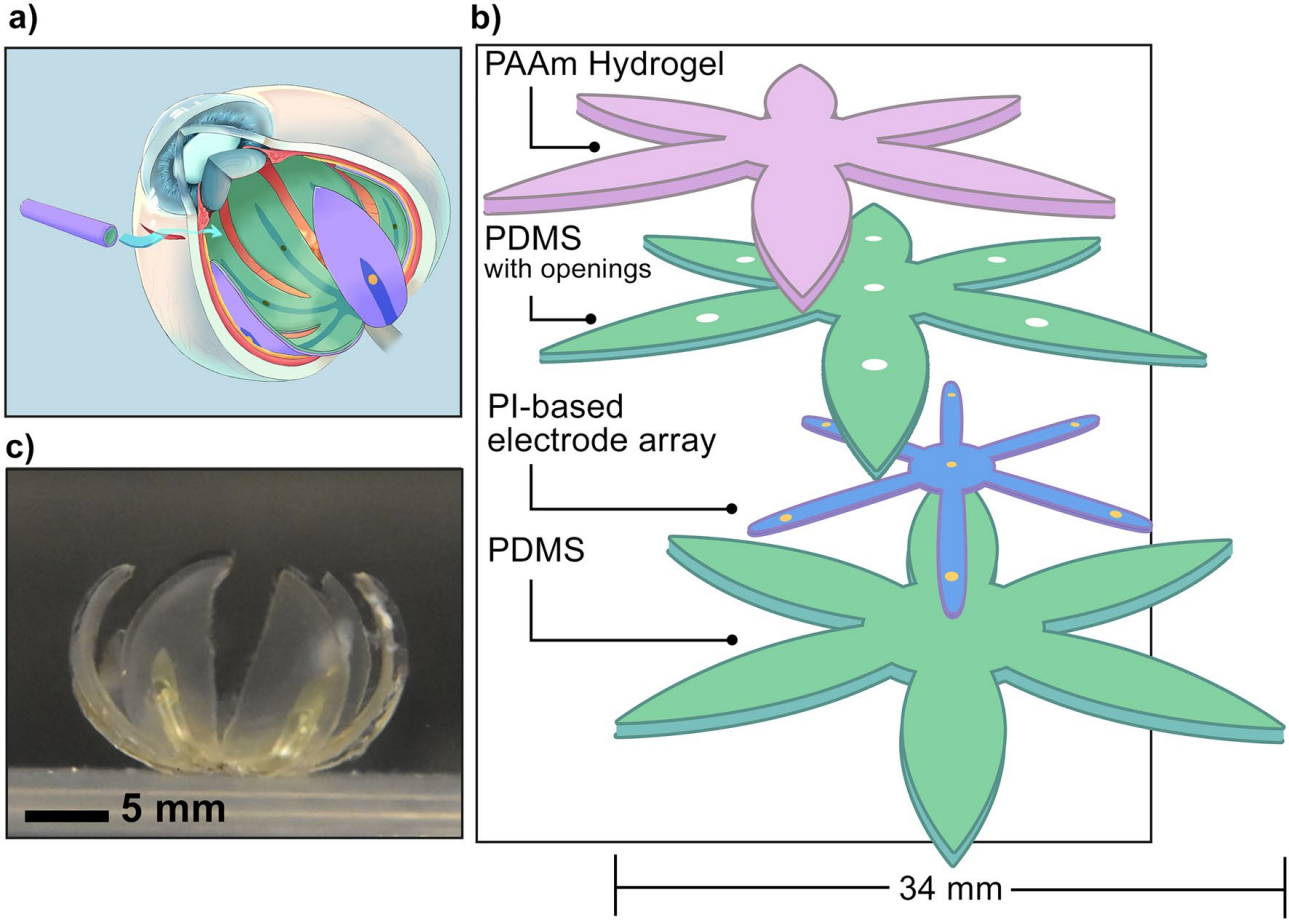
(**a**) Illustration of implanting the compact FFA into the eye. FFA unfolds with a fully swollen hydrogel layer. Notably, the illustrated metal electrodes were only an indication of the location of the electrodes, and the electrodes were opened on the same side as the hydrogel. (**b**) Layer-to-layer composition of the FFA. (**c**) A photo of the curved FFA placed on a supportive platform in 0.01 M phosphate-buffered saline (PBS).

The FFA consists of a polyimide-based electrode array sandwiched between two layers of PDMS, combined with a hydrogel layer on one side (Figure 1b). The design of the device was 6-petal flower shape with a central circular area of 6 mm (Figure 1), to approximate the wrapping of a flat surface into a sphere^34^. The diameter of the flower from a petal tip to the opposite petal tip was 34 mm (when laid flat), which would span a visual field of 113.9°^10^ in humans. Seven electrodes with a diameter of 460 μm were patterned on the substrate (1 in the center of the array and 1 on each petal) and placed to cover a visual field of 57°. The electrodes were coated, by electrodeposition, with Pt/Ir alloy to improve the electrical performance of the array^35^.

First, we deposited and patterned gold microelectrodes, conducting lines, and connection pads on polyimide. A top layer of polyimide was selectively opened to uncover microelectrodes and connections pads while insulating conducting line. The total thickness of the polyimide device is around 6 μm. Then we sandwiched the polyimide-based electrode array between two layers of PDMS, reaching a thickness of around 250 μm for all layers combined. Polyimide (PI), widely used in neural interfaces, provides a high yield of well-insulated and functional electrodes. PDMS, on the other hand, functions as both the mechanical support and the direct interface with the hydrogel. Different surface treatments are reported to promote bonding between sandwiched PI and PDMS, including oxygen plasma^30^, using poly(methyl methacrylate) (PMMA)^36^, and print transferring PI to PDMS^37^. We used oxygen plasma to treat the surface of the two layers of PDMS and laminated them to form a strong bond^38^. Because the area of the PI component was much smaller than the PDMS contact surface, the PI-based device was enveloped tightly between the two layers of PDMS, without a process to promote adhesion between PI and PDMS. Openings were created in one PDMS layer to align with the electrode sites. To avoid unintentional blocking of the electrode sites by PDMS due to misalignment, the openings in the PDMS layer were larger than the dimension of the electrode site. Since PI insulation defined the electrode site, PDMS opening size had no effect on electrode properties.

To match the device curvature to the retinal curvature and reduce electrode-to-tissue distance, the top layer of the PDMS was coated with a polyacrylamide (PAAm) hydrogel layer^21^. The transparency of PDMS allowed the UV light to pass and cure the hydrogel. Immersion in water induced swelling of the PAAm hydrogel while the volume of PDMS remained unchanged. Since the hydrogel and PDMS were bonded together at one interface, residual stress on this interface due to hydrogel swelling induced the FFA to form a 3D curvature once implanted in the eye as schematically illustrated in Figure 1a. Ideally, the radius of curvature of the device should be around 11–12.5 mm to fit the retina based on the ocular axial length of human eyes being around 22–25 mm^39^. Figure 1c shows a device with a radius of curvatures of 15.63 mm in 0.01 M PBS.

### Electrical performance evaluation

**Figure 2:**
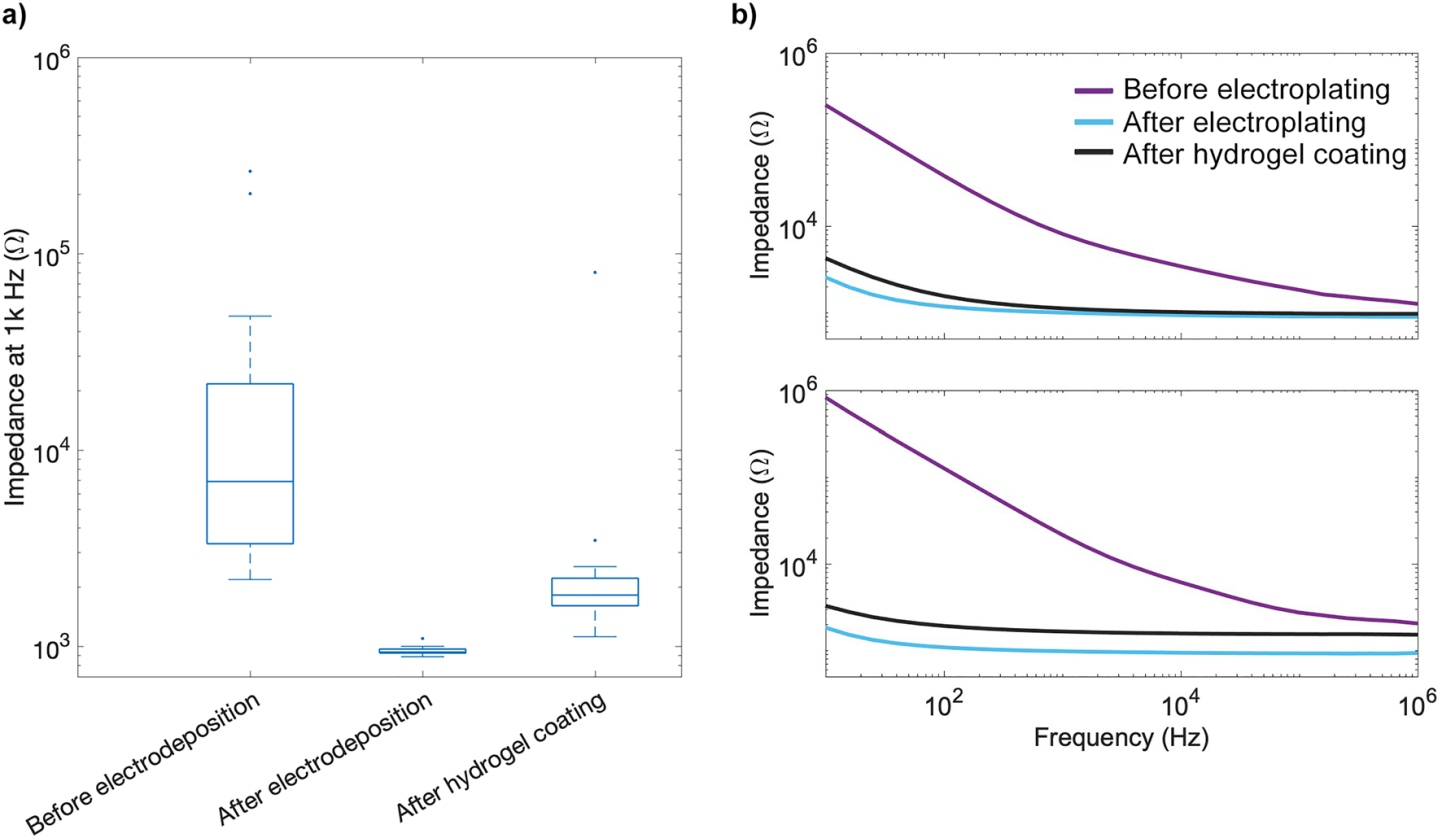
Impedance characterization of electrodes. (**a**) A box-and-whisker plot shows the distribution of impedance of the electrodes at 1 kHz (14 electrodes in total). The box represents the range of data between the first and the third quartile, and the whiskers represents the maximum and minimum data points without outliers (labeled in dots). The solid line inside the box represents the median value. (**b**) Two representative EIS trends of two out of the 14 electrodes.

Two surface modifications were performed on the electrodes: Pt/Ir electrodeposition and hydrogel coating. The electrodeposition of Pt/Ir alloy on the electrode surface was expected to increase charge storage capacity and lower the electrochemical impedance of the electrodes^40,41^. PAAm hydrogel was expected to increase impedance due to an increased resistivity (vs. PBS) as the concentration of the monomer increases^42^. To assess if these expected results were seen, we evaluated 14 electrodes on 2 devices and compared impedance and cathodic charge storage capacity (CSC_*C*_) before/after the electrodeposition as well as before/after the hydrogel coating. Figure 2a shows a box plot of the impedance at 1 kHz of 14 electrodes. Before the Pt/Ir electrodeposition, the average impedance at 1 kHz of the 14 electrodes was measured before electrodeposition as 42.92 ± 82.06 kΩ, decreasing to 0.95 ± 0.05 kΩ after electrodeposition, and reaching a value of 7.50 ± 20.95 kΩ after hydrogel coating. As expected, the electrodeposition process lowered the impedance of the electrodes after electrodeposition which slightly increased after hydrogel coating, but impedance of electrodes after hydrogel coating was still lower than naïve electrodes. The large variation of the impedance of naïve electrodes was induced by contaminations on the electrode surfaces deposited during the fabrication process as well as after scanning electron microscopy (SEM) inspection of the array between different fabrication steps. Two Electrochemical Impedance Spectroscopy (EIS) trends of representative electrodes show that some electrodes had a higher increase of the impedance at high frequency than other electrodes after hydrogel coating, suggesting increased resistivity in the conducting path due to the hydrogel coating (Figure 2b). However, the two example EIS plots show slightly different behavior. In one case there appears to be an upward shift, consistent with an increase in series resistance while the other EIS plot shows a low frequency increase only, suggesting that the electrochemical surface area was slightly diminished by hydrogel coating, but the geometric area and series resistance were unchanged. Visual inspection of the hydrogel coated electrodes under a microscope indicated variability in the thickness and adhesion of the hydrogel coating over the electrode sites. Lack of adhesion here is expected since the PDMS is removed around the electrode sites thus no bonding of the hydrogel near the electrodes will occur. However, the film may remain continuous.

Statistical analysis of EIS data was done with and without outliers. When including the outliers (see methods for outlier definition), a significant decrease of the impedance at 1 kHz of 14 electrodes was noted after electroplating with Pt/Ir alloy (one-tail paired t-test, *p*-value of 0.039), and no significant difference was observed after hydrogel coating (see Table 1). However, with outliers excluded and 8 electrodes analyzed, we found significant decrease in impedance after electrodeposition (one-tail paired t-test, *p*-value of 0.009) and significant increase in impedance after hydrogel coating (one-tail paired t-test, *p*-value of 5.042×10^-4^).

**Table 1:** One-tailed *p*-value using paired t-test. The impedance at 1 kHz and CSC_*C*_ are compared after sequential treatments. Evaluations are made both with outliers (14 electrodes) and without outliers (8 electrodes).

Comparison	Impedance at 1 kHz		CSC_*C*_	
	*p*	*p’*	*p*	*p’*
BE versus AE	0.039	0.009	1.997 × 10^–8^	1.115 × 10^–4^
AE versus AH	0.132	5.042 × 10^–4^	4.632 × 10^–8^	1.051 × 10^–4^

*BE* before electroplating, *AE* after electroplating, *AH* after hydrogel coating. *p*: *p*-value of all data; *p*’: *p*-value of data with outliers excluded.

**Figure 3:**
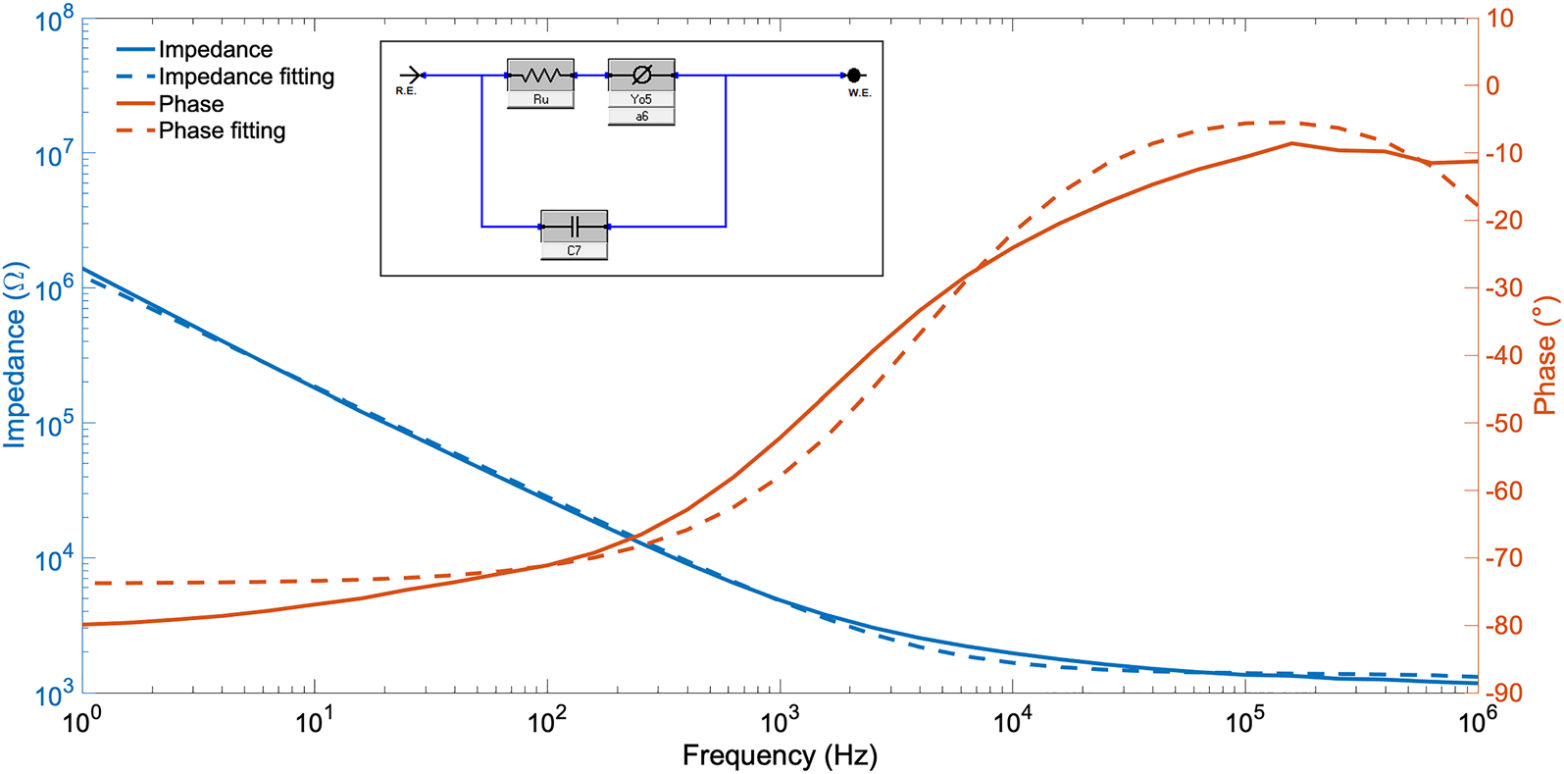
Representative plot of model fitting of the EIS measurements of one electrode before electrodeposition using an equivalent circuit model. The experimental data is reported in solid blue line for impedance and solid orange line for phase. The fitted impedance and phase are reported as blue dashed line and orange dashed lines separately. The inset shows the equivalent circuit model used to fit the experimental data.

The EIS data were fitted into an equivalent circuit model^43^ to further analyze the electrical properties of electrodes. The circuit model consisted of the following components: (1) series resistance Ru, which reflected the geometric surface area of the electrode and the resistivity of the conducting path to the counter electrode, (2) a constant phase element with admittance (Y0)and exponent (α), which reflected the electrochemical surface area and the porosity or roughness of the electrode surface and (3) a parasitic capacitance, C, which reflected instrumentation artifact caused by coupling between connecting lines. One fitting spectrum was selected as a representative example (Fig. 3). The parameters calculated by the fitting model are listed in Table 2 with standard deviation and two-tailed t-test *p*-value. Ru changed with statistical significance after electrodeposition, but only when outliers were removed. Ru before and after coating showed a large difference, but due to surface contamination, the before coating measures varied widely which led statistically significant differences in Ru only with outliers removed. Ru increased after hydrogel deposition due to the added resistivity of the hydrogel layer. Increased admittance (Y0) confirmed a lower impedance of the electrodes after electrodeposition, due to both cleaning mentioned above as well as the high surface area of Pt/Ir. When analyzing the statistical significance, we again confirmed that outliers could heavily affect the results. Paired t-test two-tail p-value suggested significance in Y0 and α before/after electroplating, and hydrogel coating did alter Y0 and α (only when outliers where removed).

**Table 2:** List of all the components extracted from the equivalent circuit model shown in Fig. 3 and two-tailed *p*-value using paired t-test, including the electrolyte resistance (Ru), the admittance (Y0), the exponent (α) of the constant phase element (CPE), and the parasitic capacitance (C). Evaluations are made both with outliers (14 electrodes) and without outliers (8 electrodes).

	Ru/Ω		Y0/(S × s^α^)		α		C/F	
BE	6216.86 ± 110.80		1.42 × 10^–7^ ± 2.23 × 10^–9^		0.784 ± 0.002		3.27 × 10^–11^ ± 2.05 × 10^–12^	
AE	911.79 ± 5.55		1.53 × 10^–5^ ± 3.47 × 10^–7^		0.862 ± 0.006		2.81 × 10^–11^ ± × 10^–12^	
AH	1583.44 ± 3848.57		1.42 × 10^–5^ ± 6.62 × 10^–7^		0.652 ± 0.007		2.58 × 10^–11^ ± 2.03 × 10^–12^	
*p*-value	*p*	*p’*	*p*	*p’*	*p*	*p’*	*P*	*p’*
BE versus AE	0.101	0.011	2.367 × 10^–7^	1.314 × 10^–5^	0.016	0.033	0.259	0.014
AE versus AH	0.001	0.001	0.257	2.628 × 10^–5^	0.002	3.780 × 10^–5^	0.547	0.255

*BE* before electroplating, *AE* after electroplating, *AH* after hydrogel coating. *p*: *p*-value of all data, *p*’: *p*-value of data with outliers excluded.

**Figure 4:**
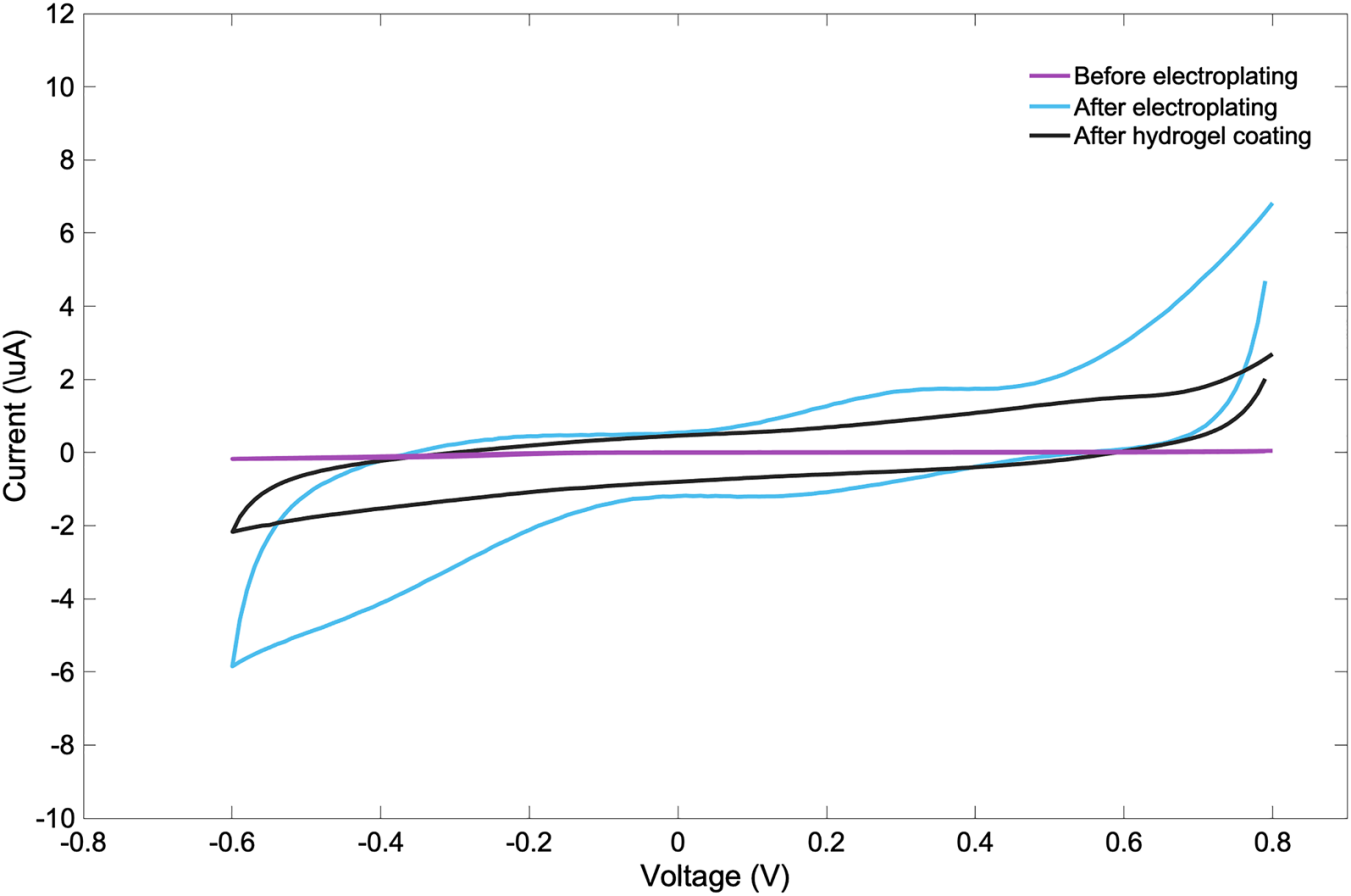
CV of one electrode before (purple) and after (blue) the Pt/Ir electrodeposition, and after hydrogel coating (black).

Cyclic voltammetry (CV) was performed on all the electrodes, and a representative CV curve of one electrode is shown in Fig. 4. The calculated CSC_*C*_ characterized the amount of charge the electrode can inject during the reversible cathodic phase of a stimulation pulse. The average CSC_*C*_ of 14 electrodes increased from 0.78 ± 0.39 mC/cm^2^ to 28.90 ± 9.31 mC/cm^2^ after electroplating with Pt/Ir, suggesting a significant improvement of the charge injection limit (one-tailed paired t-test, *p*-value of 2.00 × 10^−8^). After hydrogel coating, there was a significant reduction of the CSC_*C*_ to 13.06 ± 4.12 mC/cm^2^ (one-tailed paired t-test, *p*-value of 4.63 × 10^−8^), but still 16 times higher than naïve electrodes.

### Surface morphology of electrodes

**Figure 5:**
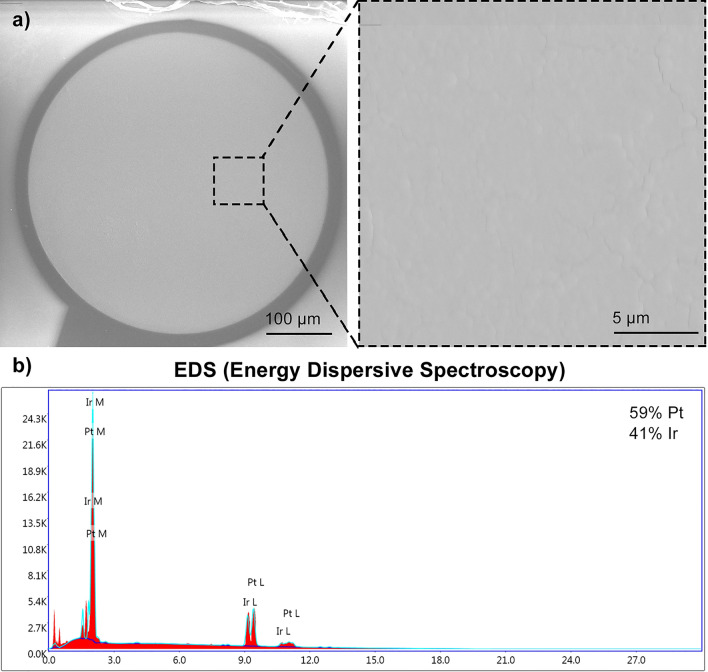
SEM image and EDS characterization of one representative electrode. (**a**) SEM image of one electrode shows the insulation of polyimide and the Pt/Ir alloy coating on the electrode surface. (**b**) Chemical spectra from the EDS map on the electrode active surface indicates two different peaks associated to Pt and Ir both at 9 keV and 11 keV. EDS map is generated using The EDAX TEAM EDS software suite. URL to the software: https://www.edax.com/-/media/ametekedax/files/eds/product_bulletins/team%20eds%20software%20suite.pdf.

SEM images were collected after the electrodeposition, and a homogenous thin film of Pt/Ir alloy was observed on the electrode surface (Fig. 5a) with some micro-cracks visible on the surface at a higher magnification. Energy dispersive spectroscopy (EDS) was done to identify the percentage of Pt and Ir on the electrode surface (Fig. 5b). A percentage of 59% for Pt and 41% of Ir was measured on one electrode, comparable with previous results^43^. SEM was done after electrodeposition but prior to hydrogel coating. Since we planned to measure impedance after SEM, we used low vacuum SEM. This mode avoided applying metal to the sample but reduced image resolution.

### Surgical simulation

**Figure 6:**
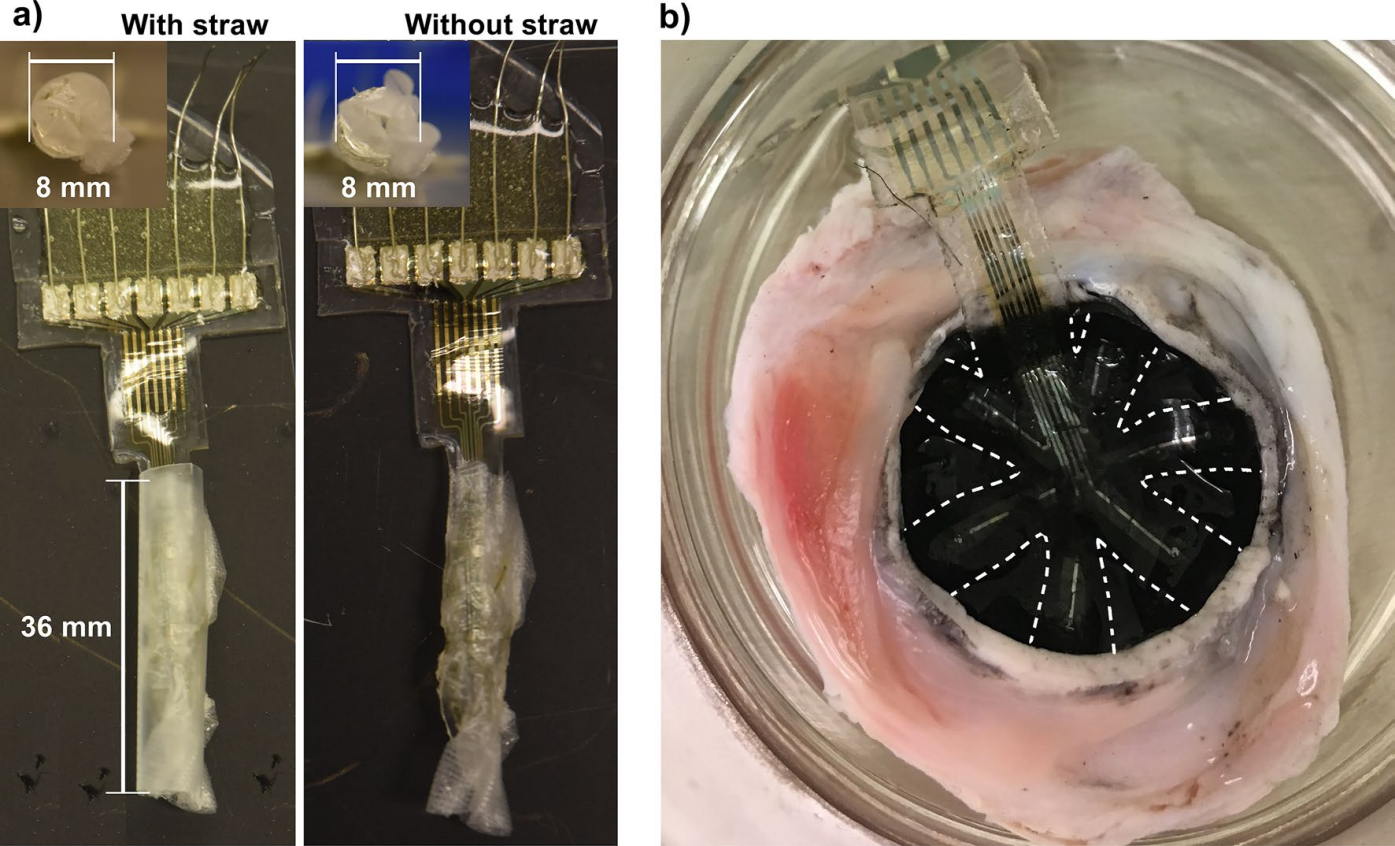
Simulation surgery to implant the multilayered device. (**a**) Preparation of the device before implantation. The multilayered prosthesis was rolled up with PVA film and placed into a straw with one side cut open. The inset is the cross-section of the rolled-up device, and the diameter is measured as 8 mm. After dehydration and the removal of the straw, the device stayed fixed and compact at a diameter of 8 mm. (**b**) Photo of the fully deployed multilayered electrode in pig eye after removal of the cornea and scleral rim. The dotted white line outlines the edges of the 6 petals.

To minimize the incision in the sclera, the FFA should be folded as compactly as possible. FFA was flexible due to the thin and narrow features of the polyimide (6 μm) and the flexibility of PDMS, while the hydrogel allowed the fixation of a temporary shape after dehydration of the multilayered prosthesis. The hydrated and flexible FFA was rolled up with water-soluble PVA film and fitted into a plastic straw, which we previously cut on one side to allow it to be flattened and then wrapped around the folded FFA. Drying the hydrogel on the FFA (at room temperature) fixed the FFA in a cylindrical shape (defined by the straw). After removing the straw, the final diameter of the rolled-up FFA was 8 mm (Figure 6a).

The cadaveric porcine eye was prepared by performing a temporary keratoprosthesis, lensectomy, and routine core vitrectomy. Placement of the keratoprosthesis (artificial cornea) and removal of the lens allowed visualization of the back of the eye. Then a 12 mm scleral incision was made parallel to the limbus at the pars plana. The FFA was then implanted in one of two fashions, each trialed in one pig eye. In the first, the FFA was inserted as a roll as described above. In the other, the FFA was not rolled, but a petal would be individually introduced through the scleral tunnel, then the FFA was rotated to allow introduction of the next petal, until all petals were introduced, termed a “Petal Walk” technique. The inserted FFA unfolded in the eye. To better visualize FFA placement within the eye, the front of the eye was removed (open-sky technique). We observed the deployed electrode array formed a continuous 3D curvature fitting to the eye and covered a wide field of the retina (Fig. 6b). After taking the electrode array out from the eye, the radius of curvature was measured 15.15 mm and the recovery rate was 96.97% compared with the curvature before implantation.

### Electrical performance evaluation after acute ex vivo implantation

**Figure 7:**
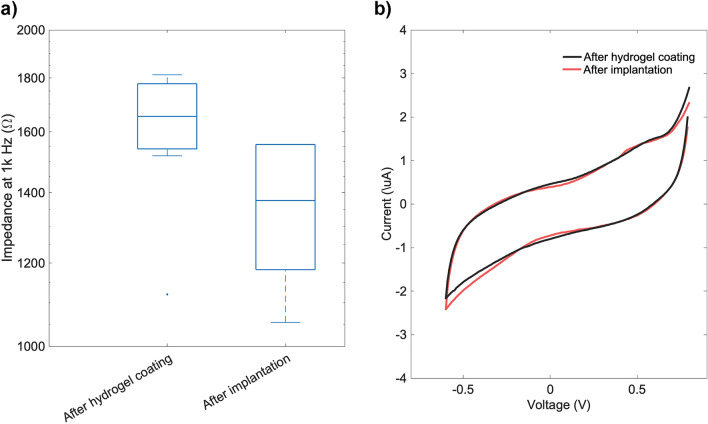
Impedance characterization of electrodes (n = 6). (**a**) A box-and-whisker plot demonstrates the impedance of electrodes at 1 kHz after hydrogel coating and after implantation. The box represents the range of data between the first and the third quartile, and the whiskers represents the maximum and minimum data points without outliers (labeled in dots). The solid line inside the box is the median value. (**b**) CV curve of one electrode shows CV after hydrogel coating in black and after implantation in red.

One FFA was extracted from the eye with intact connections. On this array, 6 out of 7 electrodes were electrically connected. The average impedance of these electrodes at 1 kHz was 2.11 ± 1.97 kΩ, measured after acute ex vivo implantation. The distribution of the impedance at 1 kHz is shown in Fig. 7a. Notably, the mean impedance decreased after the implantation. The CSC_*C*_ remained 12.62 ± 7.56 mC/cm^2^ after acute ex vivo implantation (Fig. 7b). Comparing electrodes after hydrogel coating and after implantation with outliers included, there was no statistically significant change in the impedance, the components of the model fitting (Table 3), and the CSC_*C*_. The CSC_*C*_ after acute ex vivo implantation was still significantly higher than CSC_*C*_ before electroplating, with a *p*-value of 8.90 × 10^−4^. When excluding the outliers, we found that α and C showed significant change after acute implantation.

**Table 3:** List of all the components extracted from the equivalent circuit model shown in Fig. 3 and two-tailed *p*-value using paired t-test, including the electrolyte resistance (Ru), the admittance (Y0), the exponent (α) of the constant phase element (CPE), and the parasitic capacitance (C). Evaluations are made both with outliers (6 electrodes) and without outliers (4 electrodes).

	Ru/Ω		Y0/(S × s^α^)		α		C/F	
AH	1583.44 ± 3848.57		1.42 × 10^–5^ ± 6.62 × 10^–7^		0.652 ± 0.007		2.58 × 10^–11^ ± 2.03 × 10^–12^	
AI	1617.15 ± 12.014		9.47 × 10^–6^ ± 1.90 × 10^–7^		0.679 ± 0.004		4.46 × 10^–11^ ± 2.77 × 10^–12^	
*p*-value	*p*	*p*’	*p*	*p*’	*p*	*p*’	*p*	*p*’
AH versus AI	0.438	0.053	0.080	0.061	0.567	0.038	0.248	0.001

*AH* after hydrogel coating, *AI* after implantation. *p*-values from two-tailed paired t-test were included here. *p*: *p*-value of all data; *p*’: *p*-value of data with outliers excluded.

## Discussion

We developed a full-field array for an epiretinal prosthesis that included multiple, soft materials and responded to the intraocular environment by adopting a spherical shape to match the eye. The FFA was fabricated in a planar fashion, and most of the fabrication procedures comply with cleanroom procedures. Hydrogel swelling induced 3D curvature in the FFA, fitting the curvature of the retina. The 34 mm span of the electrode array is equivalent to 113 degrees of visual angle in humans.

We used PDMS and PI for the FFA due to their long history in medical implants. Our previous study has shown that the curvature of a PDMS-PAAM bilayer can be customized by changing the formula of the hydrogel^21^. Consistent curvature between different devices will require precise control of the hydrogel layer thickness in addition to the formulation. We noted variation in curvature, which relied on a mold and manual steps for adding the hydrogel pre solution. Process refinements that can address this inconsistency include using a finer spacer^44^ and spin coating^45^. Alternative approaches to materials include a photo-sensitive polyimide coated with hydrogel, as has been done with shape-morphable sensors^46^. This would simplify the process by eliminating PDMS, but less is known about the biocompatibility of those new materials. We used benzophenone as the photoinitiator to promote bonding between PDMS and hydrogel. A rigorous protocol for removing excessive benzophenone from cured PDMS can alleviate the risks. Other biocompatible photoinitiators are also under development for future applications^47^. In addition, other materials such as thiol-ene-epoxy thermosets can be included in the fabrication process to promote longevity and maintain conformability^48^.

Electrical performance of the FFA was significantly improved by Pt/Ir electrodeposition and most of this improvement was maintained despite coating the electrodes with hydrogel. We found that contaminations from SEM and residual PDMS caused a variation of both impedance and CSC_*C*_ of the naïve electrodes, but electrodeposition effectively cleaned the electrode surface and improved the electrical properties of the electrodes due to the properties of the Pt/Ir alloy^43^. After electrodeposition, the standard deviation of the impedance at 1 kHz was reduced to 5.53%. This variation is similar to that found in a recent report on PDMS-based microelectrode impedance^49^ and the consistency is due to the repeatable electrode dimensions produced by photolithography. Hydrogel coating increased the impedance and lowered the CSC_*C*_. However, the reduced electrical capabilities may be offset by the benefit of positioning the electrodes close to the retina, where a lower stimulus threshold is expected. We also noticed that the hydrogel coating was thinner and less consistent at electrode sites and in areas where the hydrogel overlaid PI, even when the PI was covered by PDMS. It is possible that metal layers and polyimide allowed less UV light penetration compared with PDMS, reducing the polymerization and formation of the hydrogel over electrode sites. In the future, precise patterning of the hydrogel with photomasks can be explored to leave electrodes completely free of the hydrogel coating^50^.

Surgical simulation was performed using pig eyes to evaluate the process of implanting the FFA. Because FFA can be rolled up and fixed at a compact shape with a diameter of 8 mm, the sclerotomy was 12 mm, and the deployed electrode array had a diameter of 34 mm. Compared with Argus II with a 5 mm incision for a device with a width of 3 mm^51^, the size-to-incision ratio was much higher for FFA, however a 12 mm incision is unsafe as it significantly increases the risk of intraoperative hypotony, which can have significant complications. Optimizing the fabrication process to produce a thinner electrode array could further reduce the size of the folded FFA and thus a smaller sclerectomy. The water-soluble PVA film used is a commercial grade film, but other thin films are available, such as oral thin films for drug delivery^52^. Synthesized PVA films with different solubility are also reported^53^. In addition, the “Petal Walk” technique can be applied to implant the device as an alternative because of its excellent flexibility. The petal width is around 6 mm, and by “Petal Walk” technique, the size of the incision can be further reduced. The unfolded prosthesis covered most of the retina and formed a 3D configuration to conform to the retina. Notably, the size of the substrate of the electrode array covered a visual field of 113°, while the pilot study with 7 electrodes only covered 57°. For this prototype device, only a small number of electrodes were placed on the FFA. A future version of FFA could include a dense central array to provide perception of shapes and sparse peripheral electrodes, which function to provide cues towards an object of interest. This approach mimics the natural function of central and peripheral vision, but would necessitate a camera system that could detect peripheral objects. Further, the diameter of the FFA could be decreased to some degree given the proportionally decreased functional importance of increasingly eccentric retina. This prototype represents a maximized version for feasibility purposes.

Our study includes several limitations. While we achieved our goal of demonstrating a prototype FFA in an ex vivo pig eye, this necessitated the adoption of the manual process steps at some points, due to limitations in fabrication capability. In particular, patterning of PDMS holes that correspond with the electrode sites is challenging, due to known difficulty in completely removing PDMS using etchants^54,55^. We used a manual punch to cut holes in PDMS. Even then, the sandwiching process resulted in contamination of the electrodes. Approaches for patterning PDMS include laser ablation and inkjet printing, but the specialized instrumentation to pattern PDMS more precisely^49^ was not available in our facility. Alternatively, bonding hydrogel directly to polyimide can remove PDMS from the process. PDMS contamination resulted in high variance in our electrical measurements. Implantation of the FFA was performed in a cadaveric eye. Thus, we could not assess surgical complications such as bleeding and retinal injury, nor could we assess long-term biocompatibility. Such long-term tests required considerable resources and the use of large animals. Such a study should happen during preclinical testing of a retinal prosthesis that includes the FFA. To reach that point in development will require a more mature and refined fabrication process as well as extensive bench testing. Here, we have taken initial steps towards that long-term goal by creating a FFA prototype and assessing implantation in an eye model. We demonstrated a novel electrode array that can provide unprecedented coverage of the visual field and can improve function of retinal prostheses.

## Methods

### Fabrication of polyimide-based electrode array (Fig. 8a)

**Figure 8:**
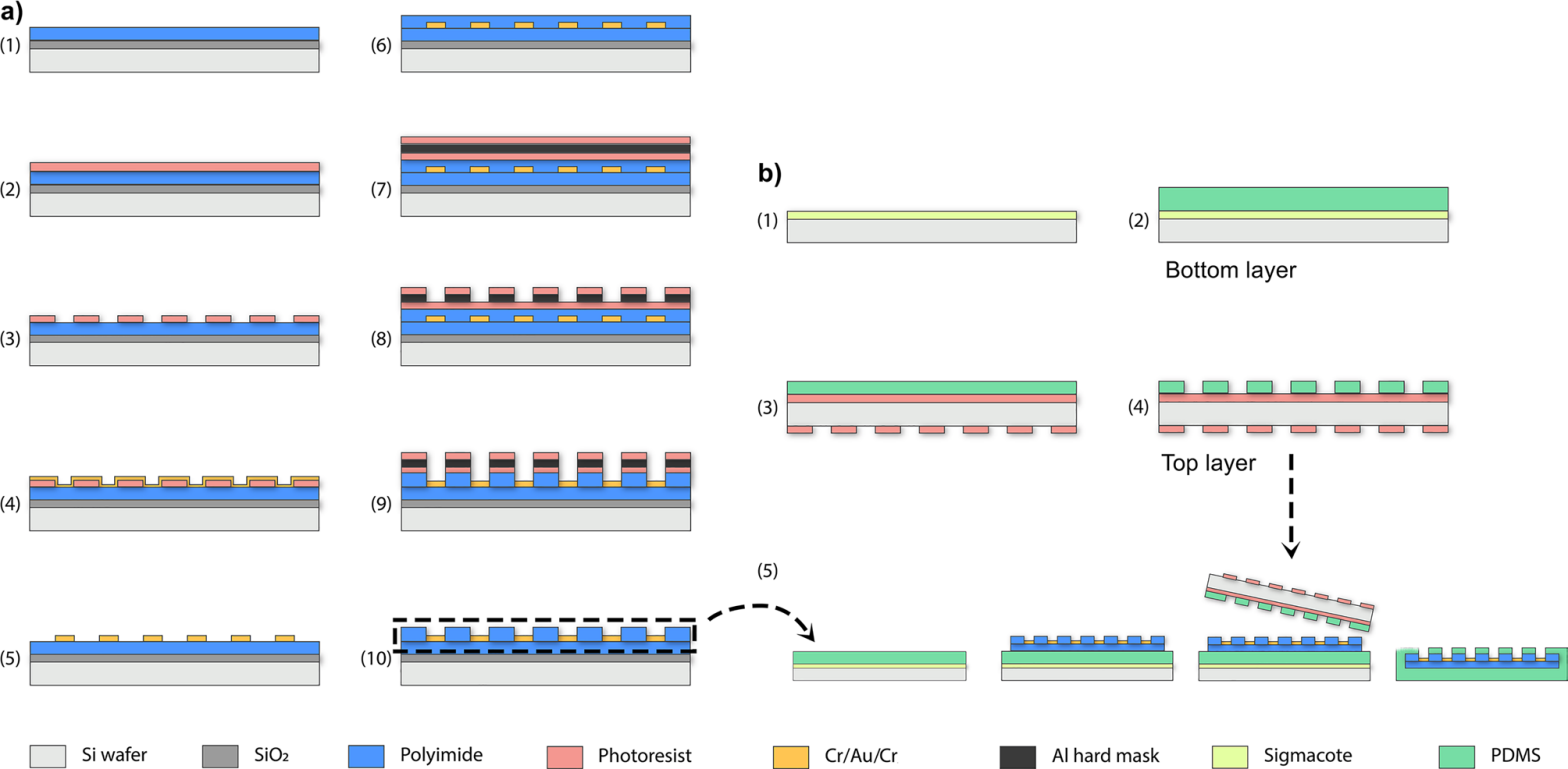
(**a**) Schematic illustration of sequential fabrication processes for polyimide-based electrode array. The bottom layer of polyimide was spin coated on the wafer and partially cured. Then, metal electrodes and conductive lines were patterned on polyimide by photolithography. Finally, the top of layer of polyimide was spin coated on top and patterned with electrode sites open. (**b**) Illustration of procedures to build sandwiched electrode array. Fabrication of the bottom layer and the top layer of PDMS with the guidance of back alignment marks. Then sandwich the polyimide-based electrode array between two layers of PDMS.

Our fabrication process involves polyimide as the direct substrate and insulation for electrodes and conductive tracks, and the polyimide-based electrode array was later sandwiched between two layers of PDMS (Fig. 8a). Polyimide is compatible with microelectromechanical systems procedures and could provide reliable electrical connections. Flexibility was achieved by making the thickness and width of the polyimide structure small.

Polyimide (PI 2611, Hitachi DuPont MicroSystems, Parlin, NJ, USA) was spin coated at 4000 rpm for 60 seconds on a Si wafer with 2 μm of SiO2 (Fig. 8a(1)). PI was soft baked at 90 °C for 10 minutes and then at 115 °C for 10 minutes on a hotplate. The partial cure of PI was done in a vacuum oven (YES PB8-2B-CP Vacuum Oven, Yield Engineering Systems, Inc., Fremont, CA, USA). Then, a photoresist (Megaposit SPR 220, DuPont, Wilmington, DE, USA) was spin coated, patterned, and developed as a mask for the following metal layers (Fig. 8a(2–3)). The wafer was cleaned with oxygen plasma (YES-CV200RFS, Yield Engineering Systems, Inc., Fremont, CA, USA). A Cr/Au/Cr layer of thickness 20 nm/200 nm/20 nm was deposited (Fig. 8a(4)). Afterward, electrodes and conductive tracks were patterned by lifting off the photoresist in acetone (Fig. 8a(5)). Then, the second layer of PI was spin coated and fully cured in the vacuum oven (Fig. 8a(6)). An Aluminum mask was patterned on the second layer of PI, and the exposed PI was completely etched by oxygen plasma (PlasmaTherm 790 RIE, Plasma-Therm LLC, Saint Petersburg, FL, USA) (Fig. 8a(7–10)). Finally, the top Cr layer is etched to expose gold and PI-based device was lifted after soaking in deionized water overnight.

### Sandwich polyimide-based electrode array between two layers of PDMS

The PI-based device was sandwiched by two layers of PDMS. The bottom layer of PDMS was around 230 μm thick and functions as the mechanical support. The top layer of PDMS was around 20 μm thick with openings for the electrodes, and this layer will be the interface to bond with a hydrogel layer (Figure 1b). The bottom layer of PDMS was spin coated on the releasing agent (Sigmacote, Sigma-Aldrich) treated glass wafer (Figure 8b (1–2)). For the top layer of PDMS, a photoresist layer was spin coated and patterned at the back of the glass wafer as alignment guidance (Figure 8b (3)). Then, a punchcutter was used to mark the area of removal on PDMS, and a razor blade was used to remove the PDMS for complete exposure of the openings (Figure 8b (4)).

Afterward, both layers of PDMS were activated with oxygen plasma (PE series, Plasma Etch Inc., Carson City, NV, USA) at 50 W for 1 minute. Then, the two layers were carefully aligned and laminated for full contact. The laminated two wafers were baked in an oven at 70 °C for 10 minutes to promote the bonding between the two layers. Finally, the top layer of PDMS was released from the wafer by soaking the laminated layers in acetone (Figure 8b (5)). Following the laser ablation to cut the outlines of the device, the sandwiched electrode array was peeled off from the bottom wafer.

### Packaging the PDMS/polyimide/PDMS electrode array

To build the connectors for electrodeposition and electrical characterizations, each contact pad on the PDMS/polyimide/PDMS electrode array was covered with silver epoxy (silver epoxy adhesive, Epo-Tek H20E Adhesive) as a conductive adhesive layer. Then a tinned copper wire (bus bar wire 28 AWG, Alpha Wire, Elizabeth, NJ, USA) adhered to the silver epoxy, and the wire was long enough to allow connection with alligator clips for electrical access to each electrode. The silver epoxy was cured at 50 °C overnight to prevent excessive thermal stress on PDMS.

### Platinum/Iridium (Pt/Ir) alloy electrodeposition

As described in prior work^43^, the plating solution was prepared with 0.2 g/L Na_3_IrCl_6_H_2_O and 0.186 g/L Na_2_PtCl_6_H_2_O in 0.1 M of nitric acid (HNO_3_). The solution was boiled until the color turned reddish, and then was cooled down to 45 °C. The temperature was kept at 45 °C throughout the deposition. A pulsing sonication (A700 Qsonica, Qsonica L.L.C. Newtown, CT, United States) at a power of 2 W (sonication on = 1 min and sonication off = 30 s) was used to maintain the constant mass transfer.

The Pt/Ir electrodeposition was done using a potential cycling method (Gamry 600 + potentiostat, Gamry Instruments, Warminster, PA, United States). A three-electrode setup was used during the potential cycling, which consists of a Pt/Ir wire with a diameter of 70 μm (A-M System, Sequim, WA, United States) as the counter electrode, an Ag/AgCl (3 M NaCl, BASi, West Lafayette, IN, United States) reference electrode, and the PDMS/polyimide/PDMS electrode array as the working electrode. The potential was scanned from -0.1 to 0.1 V with a scan rate of 200 mV/s for 1500 cycles.

### Hydrogel coating and curvature analysis

The top layer of PDMS with electrodes openings was covered by benzophenone solution (10 wt% in ethanol) for 4 min. Then, the surface was washed with methanol three times and dried in air to remove excessive benzophenone. AAm was dissolved in water to form a pre-solution with 15 wt% of AAm. The cross-linker ratio (the weight ratio of MBAM to AAm) was fixed at 3.89%, and the initiator ratio (the weight ratio of Irgacure 1173 to AAm) was fixed at 0.356%. The pre-gel solution was poured into the Teflon mold with a height of 100 μm. The benzophenone-treated PDMS surface was flipped on top of the mold. The pressure from the PDMS ensured the even distribution of the pre-solution in the mold. The pre-gel solution was cured under an ultraviolet lamp (ML-3500C, Spectroline, Westbury, NY, USA) for 10 min. After curing, the FFA was carefully peeled off along the laser-ablate outline, and the FFA was put in 0.01 M PBS, which caused the hydrogel to swell and the electrode array to form a 3D flower shape.

After the FFA swelled and reached the final curvature, a camera (D7200, Nikon, Tokyo, Japan) was used to take photos of the hydrated multilayer, and the curvature analysis was done using the digital imaging processing software Fiji and the plugin Kappa. Kappa measured the curvature of each point along the diameter spline of the electrode array, then the average of the curvature was used to represent the curvature of the electrode array.

### Electrochemical impedance spectroscopy and cyclic voltammetry

Electrochemical impedance spectroscopy (EIS) was collected using a potentiostat (Gamry Reference 600 + Potentiostat, Gamry Inc., Warminster, PA, United States). EIS was performed with a 10 mV (peak) sine wave at a frequency range of 10 Hz–1 MHz in a solution of 0.01 M PBS with the three-electrode setup. Experimental data were fitted to an equivalent circuit model, and the values of the electrolyte resistance (Ru), the admittance (Y0), the exponent (α) of the constant phase element (CPE), and the parasitic capacitance (C) were calculated^43^.

Cyclic voltammetry (CV) was measured with the three-electrode setup using the potentiostat in 0.01 M PBS. The voltage was swept from − 0.6 to 0.8 V versus Ag/AgCl at a scan rate of 50 mV/s. Cathodal charge storage capacity (CSC_*C*_) was calculated from the integration of the area of the time-dependent cathodic current curve.

### Ex vivo acute implantation surgery in pig eyes

Pig eyes were frozen and ready to be used (Animal Technologies, Inc., Tyler, TX, USA). Pig eyes were used as the eye model because of its availability and similarity to the human eye in size^56^. The FFA was carefully rolled up with polyvinyl alcohol (PVA) film (water soluble stabilizer, Superpunch, Quebec, Canada) and placed in a straw. PVA film was water soluble and facilitated the rolling process. Following overnight dehydration of the hydrogel, the electrode array was fixed at a temporary compact shape.

The porcine eye was fixed to a styrofoam block. Three DORC (EVA Phaco-Vitrectomy system, Dutch Ophthalmic USA, Inc., Exeter, NH, USA) 25 g valved trocars were placed 4 mm posterior to the limbus at 2, 4 and 10 o'clock. Then, a central 7 mm ring was measured in the cornea, and excised using curved scissors. A Landers Widefield Temporary Keratoprosthesis (Ocular Instruments, Bellevue, WA, USA) was then sutured into position using six 9-0 nylon sutures. The infusion line was inserted into the 4oc trocar, and opened to normal saline. The porcine crystalline lens was removed using a 25 g microvitrector. Then, a wide-field contact lens was placed onto the keratoprosthesis, and a core vitrectomy was performed. Once adequate vitrectomy was completed, a 90-degree peritomy was performed from 6 to 9 o'clock. Then, a 12 mm scleral wound was fashioned using a 64 blade scalpel 4 mm posterior to the limbus.

The device was implanted in one of two fashions: either a rolled insertion, where the device was rolled, grasped in non-toothed forceps, then directly driven through the scleral incision, or by a "Petal Walk" technique, where the device was not rolled, but each petal was individually introduced through the scleral tunnel. Care was taken to minimize trauma to the device during the petal walk to avoid shearing the plastic housing. With rolled insertion, it was critical to insert the device quickly, in one movement, to avoid the PVA coating from dissolving on contact with fluid and allowing the device to unroll prematurely. Once the device was inserted with either method, its conformation to the retina was observed. Then, three to four 9-0 nylon sutures were placed in simple interrupted fashion to close the sclerotomy and tested to be water-tight. Finally, the cornea and 4 mm of scleral rim were cut 360 with curved scissors to better visualize and image the deployed device in the pig eye.

### SEM and statistical analysis

SEM images were taken (Tescan Rise, Tescan Orsay Holding, Brno-Kohoutovice, Czech Republic) in a low vacuum mode at 20 kV to prevent the deposition of conductive films on top of the electrodes. The electrode was steadily adhered to an SEM stub using carbon tapes.

A paired t-test was used to compare the data before and after each of the following treatments: electrodeposition, hydrogel coating, and acute implantation. Data including the impedance of electrodes at 1 kHz, CSC_*C*_, and the parameters calculated from the circuit model were analyzed. Outliers of electrodes are defined if their impedance at 1 kHz is less than q1 – 1.5 × (q3–q1) or greater than q3 + 1.5 × (q3–q1), in which q1 and q3 are equal to the first and the third quartiles of the impedance data based on all the 14 electrodes. Notably, because of the existence of the outliers, we presented *p-*value from the paired t-test both with and without outliers in most of the analyses, and the alpha level was 0.05. Sample sizes with or without the outliers are described case by case in the results section.

## Data Availability

The datasets generated during and/or analyzed during the current study are available from the corresponding author on reasonable request.

## References

[1] Borton D, Micera S, Millán JDR, Courtine G (2013). Personalized neuroprosthetics. Sci. Transl. Med..

[2] Rizzo S (2019). Assessment of postoperative morphologic retinal changes by optical coherence tomography in recipients of an electronic retinal prosthesis implant. JAMA Ophthalmol..

[3] Ray A, Chan LLH, Gonzalez A, Humayun MS, Weiland JD (2011). Impedance as a method to sense proximity at the electrode-retina interface. IEEE Trans. Neural Syst. Rehabil. Eng..

[4] Berson EL (1993). Retinitis pigmentosa: The friedenwald lecture. Investig. Ophthalmol. Vis. Sci..

[5] Weiland JD, Liu W, Humayun MS (2005). Retinal prosthesis. Annu. Rev. Biomed. Eng..

[6] Borda, E. & Ghezzi, D. Progress in Biomedical Engineering Advances in visual prostheses : engineering and biological challenges Progress in Biomedical Engineering OPEN ACCESS Advances in visual prostheses : engineering and biological challenges. (2022).

[7] Herse P (2005). Retinitis pigmentosa: Visual function and multidisciplinary management. Clin. Exp. Optom..

[8] Ayton LN (2020). An update on retinal prostheses. Clin. Neurophysiol..

[9] Vagni P (2022). POLYRETINA restores light responses in vivo in blind Göttingen minipigs. Nat. Commun..

[10] Ameri H (2009). Toward a wide-field retinal prosthesis. J. Neural Eng..

[11] Liu, Y. *et al.* Parylene origami structure for introcular implantation. *2013 Transducers Eurosensors XXVII 17th Int. Conf. Solid-State Sensors, Actuators Microsystems, TRANSDUCERS EUROSENSORS 2013* 1549–1552 (2013). doi:10.1109/Transducers.2013.6627077

[12] Ferlauto L (2018). Design and validation of a foldable and photovoltaic wide-field epiretinal prosthesis. Nat. Commun..

[13] Waschkowski F (2014). Development of very large electrode arrays for epiretinal stimulation (VLARS). Biomed. Eng. Online.

[14] Wang J (2019). Self-unfolding flexible microelectrode arrays based on shape memory polymers. Adv. Mater. Technol..

[15] Ware T (2013). Thiol-click chemistries for responsive neural interfaces. Macromol. Biosci..

[16] Macron J, Gerratt AP, Lacour SP (2019). Thin hydrogel–elastomer multilayer encapsulation for soft electronics. Adv. Mater. Technol..

[17] Zhang Y (2019). Climbing-inspired twining electrodes using shape memory for peripheral nerve stimulation and recording. Sci. Adv..

[18] Hager MD, Bode S, Weber C, Schubert US (2015). Shape memory polymers: Past, present and future developments. Prog. Polym. Sci..

[19] Jeon SJ, Hauser AW, Hayward RC (2017). Shape-morphing materials from stimuli-responsive hydrogel hybrids. Acc. Chem. Res..

[20] Magdanz V, Stoychev G, Ionov L, Sanchez S, Schmidt OG (2014). Stimuli-responsive microjets with reconfigurable shape. Angew. Chemie - Int. Ed..

[21] Zhou M, Kang DH, Kim J, Weiland JD (2020). Shape morphable hydrogel/elastomer bilayer for implanted retinal electronics. Micromachines.

[22] Yuk H, Lu B, Zhao X (2019). Hydrogel bioelectronics. Chem. Soc. Rev..

[23] Liu X, Liu J, Lin S, Zhao X (2020). Hydrogel machines. Mater. Today.

[24] Victor A, Ribeiro J, Araújo F (2019). Study of PDMS characterization and its applications in biomedicine: A review. J. Mech. Eng. Biomech..

[25] Rivnay J, Wang H, Fenno L, Deisseroth K, Malliaras GG (2017). Next-generation probes, particles, and proteins for neural interfacing. Sci. Adv..

[26] Schiavone G (2020). Soft, implantable bioelectronic interfaces for translational research. Adv. Mater..

[27] Yan D (2019). Ultracompliant carbon nanotube direct bladder device. Adv. Healthc. Mater..

[28] Li X (2021). PDMS-parylene hybrid, flexible micro-ECoG electrode array for spatiotemporal mapping of epileptic electrophysiological activity from multicortical brain regions. ACS Appl. Bio Mater..

[29] Lee KY (2020). Development of a polydimethylsiloxane-based electrode array for electrocorticography. Adv. Mater. Interfaces.

[30] Moon JH (2010). Wearable polyimide-PDMS electrodes for intrabody communication. J. Micromechanics Microengineering.

[31] Liu Y (2019). Soft and elastic hydrogel-based microelectronics for localized low-voltage neuromodulation. Nat. Biomed. Eng..

[32] Yuk H, Zhang T, Parada GA, Liu X, Zhao X (2016). Skin-inspired hydrogel-elastomer hybrids with robust interfaces and functional microstructures. Nat. Commun..

[33] Lu Y (2009). Poly(vinyl alcohol)/poly(acrylic acid) hydrogel coatings for improving electrode-neural tissue interface. Biomaterials.

[34] Demaine ED, Demaine ML, Iacono J, Langerman S (2009). Wrapping spheres with flat paper. Comput. Geom. Theory Appl..

[35] Della Valle E, Welle EJ, Chestek CA, Weiland JD (2021). Compositional and morphological properties of platinum-iridium electrodeposited on carbon fiber microelectrodes. J. Neural Eng..

[36] Rezaei S, Xu Y, Pang SW (2019). Control of neural probe shank flexibility by fluidic pressure in embedded microchannel using PDMS/PI hybrid substrate. PLoS ONE.

[37] Pu Z (2019). A flexible precise volume sensor based on metal-on-polyimide electrodes sandwiched by PDMS channel for microfluidic systems. Microfluid. Nanofluidics.

[38] Eddings MA, Johnson MA, Gale BK (2008). Determining the optimal PDMS-PDMS bonding technique for microfluidic devices. J. Micromechanics Microengineering.

[39] Bhardwaj V, Rajeshbhai GP (2013). Axial length, anterior chamber depth-a study in different age groups and refractive errors. J. Clin. Diagnostic Res..

[40] Petrossians A, Whalen JJ, Weiland JD, Mansfeld F (2011). Electrodeposition and characterization of thin-film platinum-iridium alloys for biological interfaces. J. Electrochem. Soc..

[41] Cassar IR (2019). Electrodeposited platinum-iridium coating improves in vivo recording performance of chronically implanted microelectrode arrays. Biomaterials.

[42] Holder DS, Khan A (1994). Use of polyacrylamide gels in a saline-filled tank to determine the linearity of the sheffield mark 1 electrical impedance tomography (EIT) system in measuring impedance disturbances. Physiol. Meas..

[43] della Valle E (2021). Electrodeposited patinum iridium enables microstimulation with carbon fiber electrodes. Front. Nanotechnol..

[44] Yoon J, Cai S, Suo Z, Hayward RC (2010). Poroelastic swelling kinetics of thin hydrogel layers: Comparison of theory and experiment. Soft Matter.

[45] Chollet B (2016). Multiscale surface-attached hydrogel thin films with tailored architecture. ACS Appl. Mater. Interfaces.

[46] Karnaushenko D (2015). Self-assembled on-chip-integrated giant magneto-impedance sensorics. Adv. Mater..

[47] Taschner R, Gauss P, Knaack P, Liska R (2020). Biocompatible photoinitiators based on poly-α-ketoesters. J. Polym. Sci..

[48] Borda E, Medagoda DI, Airaghi Leccardi MJI, Zollinger EG, Ghezzi D (2023). Conformable neural interface based on off-stoichiometry thiol-ene-epoxy thermosets. Biomaterials.

[49] Minev IR (2015). Electronic dura mater for long-term multimodal neural interfaces. Science..

[50] Yanagawa F, Sugiura S, Kanamori T (2016). Hydrogel microfabrication technology toward three dimensional tissue engineering. Regen. Ther..

[51] Cheng DL, Greenberg PB, Borton DA (2017). Advances in retinal prosthetic research: A systematic review of engineering and clinical characteristics of current prosthetic initiatives. Curr. Eye Res..

[52] Sevinç Özakar R, Özakar E (2021). Current overview of oral thin films. Turkish J. Pharm. Sci..

[53] Chan LW, Hao JS, Heng PWS (1999). Evaluation of permeability and mechanical properties of composite polyvinyl alcohol films. Chem. Pharm. Bull..

[54] Garra J (2002). Dry etching of polydimethylsiloxane for microfluidic systems. J. Vac. Sci Technol. A Vac. Surf. Film..

[55] Balakrisnan B, Patil S, Smela E (2009). Patterning PDMS using a combination of wet and dry etching. J. Micromechanics Microengineering.

[56] Ratanapakorn T, Ameri H, Humayun MS, Weiland JD (2006). Enucleated eye model for intraocular retinal prosthesis implantation. Ophthalmic Surg. Lasers Imaging.

